# Isolation of deoxynivalenol-transforming bacteria from the chicken intestines using the approach of PCR-DGGE guided microbial selection

**DOI:** 10.1186/1471-2180-10-182

**Published:** 2010-06-24

**Authors:** Hai Yu, Ting Zhou, Jianhua Gong, Christopher Young, Xiaojun Su, Xiu-Zhen Li, Honghui Zhu, Rong Tsao, Raymond Yang

**Affiliations:** 1 Guelph Food Research Centre, Agriculture and Agri-Food Canada, 93 Stone Road West, Guelph, Ontario, N1G 5C9, Canada; 2 Key Laboratory for Crop Germplasm Innovation and Utilization of Hunan Province, Hunan Agricultural University, Changsha, 410128, China

## Abstract

**Background:**

Contamination of grains with trichothecene mycotoxins, especially deoxynivalenol (DON), has been an ongoing problem for Canada and many other countries. Mycotoxin contamination creates food safety risks, reduces grain market values, threatens livestock industries, and limits agricultural produce exports. DON is a secondary metabolite produced by some *Fusarium *species of fungi. To date, there is a lack of effective and economical methods to significantly reduce the levels of trichothecene mycotoxins in food and feed, including the efforts to breed *Fusarium *pathogen-resistant crops and chemical/physical treatments to remove the mycotoxins. Biological approaches, such as the use of microorganisms to convert the toxins to non- or less toxic compounds, have become a preferred choice recently due to their high specificity, efficacy, and environmental soundness. However, such approaches are often limited by the availability of microbial agents with the ability to detoxify the mycotoxins. In the present study, an approach with PCR-DGGE guided microbial selection was developed and used to isolate DON -transforming bacteria from chicken intestines, which resulted in the successful isolation of several bacterial isolates that demonstrated the function to transform DON to its de-epoxy form, deepoxy-4-deoxynivalenol (DOM-1), a product much less toxic than DON.

**Results:**

The use of conventional microbiological selection strategies guided by PCR-DGGE (denaturing gradient gel electrophoresis) bacterial profiles for isolating DON-transforming bacteria has significantly increased the efficiency of the bacterial selection. Ten isolates were identified and isolated from chicken intestines. They were all able to transform DON to DOM-1. Most isolates were potent in transforming DON and the activity was stable during subculturing. Sequence data of partial 16S rRNA genes indicate that the ten isolates belong to four different bacterial groups, Clostridiales, *Anaerofilum*, *Collinsella*, and *Bacillus*.

**Conclusions:**

The approach with PCR-DGGE guided microbial selection was effective in isolating DON-transforming bacteria and the obtained bacterial isolates were able to transform DON.

## Background

Deoxynivalenol (DON; vomitoxin) is a secondary metabolite produced by some *Fusarium *species of fungi. DON belongs to the trichothecene group of mycotoxins characterized by the 12,13-epoxy-trichothec-9-ene ring system. It has been shown that the 12,13-epoxide group on the trichothecene nucleus of DON is mainly responsible for its toxicity [1,2]. The toxin causes clinical symptoms including feed refusal, vomiting, lesions in the gastrointestinal tract, immunosuppression and lack of muscle coordination in domestic animals [2-4]. DON contamination often occurs when weather is conducive to the infection of cereal crops by *Fusarium *fungi and is commonly found worldwide on corn, wheat, barley, and other grains. Contamination of grains by DON poses an increasingly serious threat to livestock production and human health. Despite a plethora of information regarding the biochemistry, toxicity, and modes of action of mycotoxins, it still remains a challenge to control/eradicate DON either pre- or post- harvest [5]. The industries are facing an even greater challenge due to the increased incidence of *Fusarium *ear rot of corn and the competition for corn from the emerging biofuel industry [6]. Therefore, effective methods to control mycotoxin contamination are urgently needed.

The prevention of mycotoxin production and detoxification of mycotoxins are the two main strategies for control of mycotoxin contamination. While physical and chemical techniques have been largely used to detoxify DON, breeding for *Fusarium-*resistant plants and preharvest use of fungicides are the main strategies for the prevention [7]. Biological detoxification has also been a choice for postharvest treatment because of its advantages in efficiency, specificity, and environmental soundness. A de-epoxy metabolite of DON, resulting from enzymatic reduction of the 12,13-epoxy-group to a diene, was identified from rat urine and faeces and first described by Yoshizawa *et al. *[8]. The de-epoxy DON, called dE-DON or DOM-1 in the literature, has been proven to be much less toxic than DON [2,9,10]. Biotransformation of DON by microbial cells or enzymes is particularly attractive [11-13]. In the past two and half decades, transformation of DON by mixed microorganisms from animal intestines has been studied [5]. One significant study showed that DON incubated *in vitro *with the contents of the large intestine of chicken (CLIC) disappeared within 24 hr [14]. Subsequently, it was confirmed that microorganisms in CLIC were able to completely transform DON to DOM-1 and the activity was retained through 6 serial subcultures [12]. The transformation of DON and the significant reduction in its toxicity was demonstrated by a pig feeding experiment [9]. Both *in vitro *and *in vivo *studies have also shown that DON can be transformed to DOM-1 by intestinal microorganisms of other animal species including cow, rat, sheep, and pig [10,15-18]. Although mixed microorganisms from animal intestines often demonstrated the ability to transform DON to DOM-1, isolation of DON-transforming microorganisms to a pure culture has been a great challenge. There have been only a few reports on DON transformation by a pure bacterial culture [5]; only one of these cases thus far, *Eubacterium *sp., isolated from the rumen [19], has been systematically studied. It appears that the lack of pure cultures of transforming bacteria has limited the full implementation of biological detoxification strategies. The present research was conducted to select DON-transforming bacteria from the chicken intestines with potential application in the management of mycotoxin risks.

## Results

### *In vivo *enrichment

The effect of feeding DON-contaminated wheat on the enrichment of DON-transforming bacteria in the chicken intestines was initially investigated. Digesta samples from the large intestine (LIC) of layers fed DON-contaminated wheat were able to completely transform DON in the medium to DOM-1 after incubation. However, only 80% DON on average (standard deviation = 16.4) was transformed by the digesta samples from the layers fed clean wheat. Similar results were obtained with the digesta samples from the small intestine (SIC).

### Effect of media

Different media were examined initially for their effect on the activity of DON transformation and also on the bacterial growth of digesta samples. Among the tested media including AIM, AIM+CecExt, L10, MRS, RB, VL, and DAM, only L10 and AIM+CecExt fully supported the transformation of DON to DOM-1 (100%). While bacterial cultures could be rapidly established in L10 broth, the growth of bacteria in AIM + CecExt was minimal. These two media were therefore used for subsequent selection for DON-transforming bacteria, depending on the aim of particular experiments.

### DON-transforming activity of digesta samples and their subcultures

The level of DON-transforming activity in the digesta samples collected from the crop, small and large intestines of chickens fed DON-contaminated or clean wheat was determined. Among 12 chickens examined, 92% LIC (11 out of 12) and 50% SIC (5 out of 10) samples transformed DON to DOM-1 completely after 72 hr incubation. However, only 25% (1 out of 4) samples from the chicken crop demonstrated a partial activity in transforming DON to DOM-1 (conversion = 26%) after 72 hr incubation.

**Figure 1:**
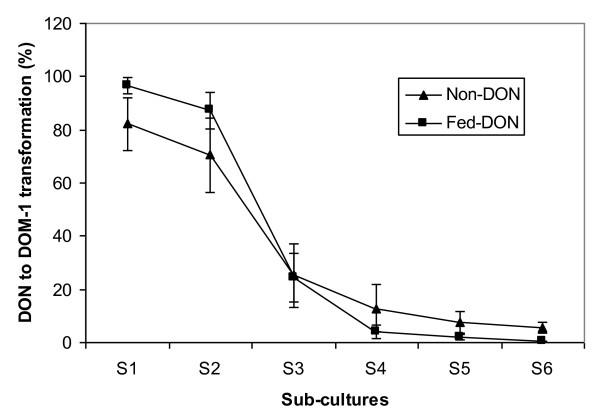
**Transformation of DON to DOM-1 by the subcultures of digesta samples **. The digesta samples were from the large intestine of chickens fed clean or DON-contaminated wheat (10 μg g^-1 ^DON) during the *in vivo *enrichment experiment. The subcultures were grown in L10 broth containing 100 μg ml^-1 ^DON. Each subculture was incubated for 72 hours. n = 6.

The LIC digesta samples collected from the chickens fed DON-contaminated or clean wheat were also examined for their activity of DON transformation during subculturing (6 passages, 72 hr per subculture) in L10 broth. The first two subcultures retained a high activity in transforming DON. However, the activity declined significantly from the third passage on (Fig. 1).

### Selection for DON-transforming bacteria

**Figure 2:**
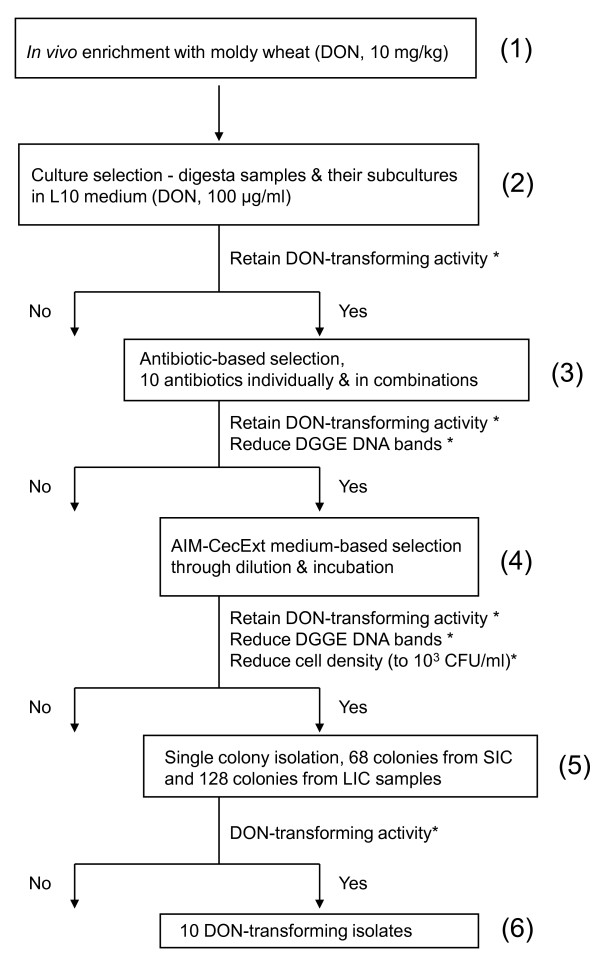
**Flow chart showing the process of selection for chicken intestinal bacteria with the ability to transform DON **. *Selection criteria used in each step of the selection. Numbers in the parentheses indicate particular steps in the selection.

When individual antibiotics were tested for bacterial selection (Step 3 in Fig. 2), virginiamycin, lincomycin, and tylosin showed no detrimental effect on either the activity of DON transformation or bacterial growth of the start cultures at all tested concentrations (Table 1). However, a similar effect was observed only at the low concentration (5 μg ml^-1^) of streptomycin, penicillin G, and salinomycin. Different combinations of these antibiotics were then investigated for their effect on supporting the activity of DON transformation and the growth of bacterial cells. Only one combination containing virginiamycin (20 μg ml^-1^), lincomycin (60 μg ml^-1^), and salinomycin (5 μg ml^-1^) significantly reduced the growth of bacterial cells without detrimental effect on the DON-transforming activity. Hence, the cultures selected through this combination were used for further selection by the AIM+CecExt medium.

**Table 1:** Effects of antibiotics on the growth and DON-transforming activity of bacteria from the large (LIC) or small (SIC) intestine.

Antibiotics	Final concen (μg/mL)	LIC-S2		LIC-S3		SIC-S2		SIC-S3	
					
		Growth	DON to DOM-1 (%)			Growth	DON to DOM-1 (%)		
No antibiotic	0	+++	100.0	N/A		+++	100.0	+++	100.0
Streptomycin	100	+++	49.3	+++	25.6	+++	44.3	+++	5.8
	50	+++	100.0	+++	30.8	+++	48.7	+++	11.4
	5	+++	100.0	+++	100.0	+++	100.0	+++	100.0
Gentamicin	80	+++	18.1	+++	6.0	++	44.0	+++	7.1
	40	+++	23.5	+++	6.5	+++	44.8	+++	7.4
	5	+++	100.0	+++	22.5	+++	46.5	+++	6.8
Bacitracin	60	++	16.2	++	0.0	+++	45.0	+++	8.0
	30	++	16.1	++	2.5	+++	45.0	+++	8.8
	5	+++	15.8	+++	3.9	+++	47.0	+++	11.9
No antibiotic	0	+++	100.0	+++	100.0	+++	100.0	+++	100.0
Penicillin G	100	+	12.1	+++	1.5	++	100.0	+	35.5
	50	+	12.7	+++	7.4	++	100.0	+	44.1
	5	++	100.0	+++	100.0	+++	100.0	+++	100.0
Virginiamycin	20	+++	100.0	+++	100.0	+++	100.0	+++	100.0
	10	+++	100.0	+++	100.0	+++	100.0	+++	100.0
	5	+++	100.0	+++	100.0	+++	100.0	+++	100.0
Lincomycin hydrochloride	60	+++	100.0	+++	100.0	+++	31.3	+++	3.6
	30	+++	100.0	+++	100.0	+++	100.0	+++	100.0
	5	+++	100.0	+++	100.0	+++	47.3	+++	100.0
No antibiotic	0	+++	100.0	+++	100.0	+++	100.0	+++	100.0
Salinomycin	80	+++	16.7	+++	2.0	+++	55.2	+++	8.9
	40	+++	18.0	+++	4.0	+++	89.2	+++	80.9
	5	+++	16.8	+++	100.0	+++	100.0	+++	100.0
Vancomycin	30	+++	15.9	+++	2.5	++	46.2	+++	9.6
	15	+++	15.0	+++	2.2	++	44.9	+++	10.5
	5	+++	38.5	+++	13.2	++	46.8	+++	9.7
Carbadox	50	+++	16.4	++	3.5	++	27.7	+++	3.9
	25	+++	100.0	+++	45.2	+++	100.0	+++	52.7
	5	+++	100.0	+++	78.7	+++	100.0	+++	100.0
Tylosin	80	+++	100.0	+++	100.0	+++	100.0	+++	79.4
	40	+++	100.0	+++	100.0	+++	100.0	+++	92.2
	5	+++	100.0	+++	94.5	+++	100.0	+++	100.0

Note: LIC-S2 and SIC-S2 mean inoculum from the first sub-culture of the large intestinal digesta or small intestinal digesta, respectively.

+ means slight growth; ++ moderate growth; +++ vigorous growth

**Figure 3:**
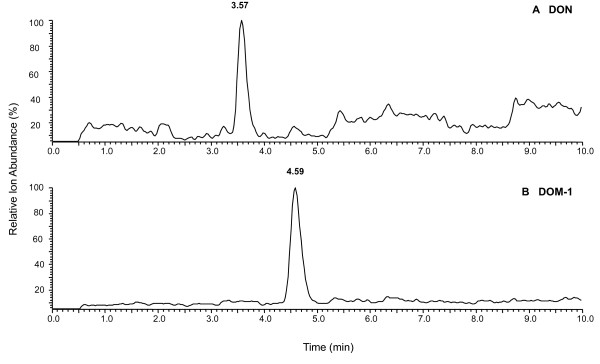
**LC-MS chromatograms showing the biotransformation of DON to DOM-1 **. **A) **DON (100 μg ml^-1^) in L10 broth without any bacterial inoculum after 72 hr incubation. Selected ion monitoring at *m/z *231, 249, 267, 279, and 297. **B) **Transformation of DON (100 μg ml^-1^) to DOM-1 in L10 broth inoculated with isolate LS100 after 72 hr incubation. Selected ion monitoring at *m/z *215, 233, 245, 251, 263, and 281.

The previously selected cultures were diluted 10-fold in series, inoculated in the AIM+CecExt medium, incubated for 72 hr, and then examined for DON-transforming activity (Step 4 in Fig. 2). Among the serially diluted cultures (from 10^-1 ^to 10^-5^), the diluted cultures in 10^-1^, 10^-2^, or 10^-3 ^all completely transformed DON to DOM-1 in the medium. However, the diluted cultures in 10^-4 ^and 10^-5 ^demonstrated a partial activity of DON transformation with 44 and 24% of DON transformed to DOM-1, respectively. The process was repeated until the cultures had their cell density reduced to 10^3 ^CFU ml^-1^, but still retained full activity of DON transformation prior to single colony isolation on L10 agar. Sixty eight and 128 single colonies were isolated from the diluted SIC and LIC cultures, respectively, and ten isolates (representing approximately 5% of the colonies examined) were found to be capable of transforming DON to DOM-1 (Fig. 3). One of the isolates was from the small intestine and the remaining from the large intestine.

**Figure 4:**
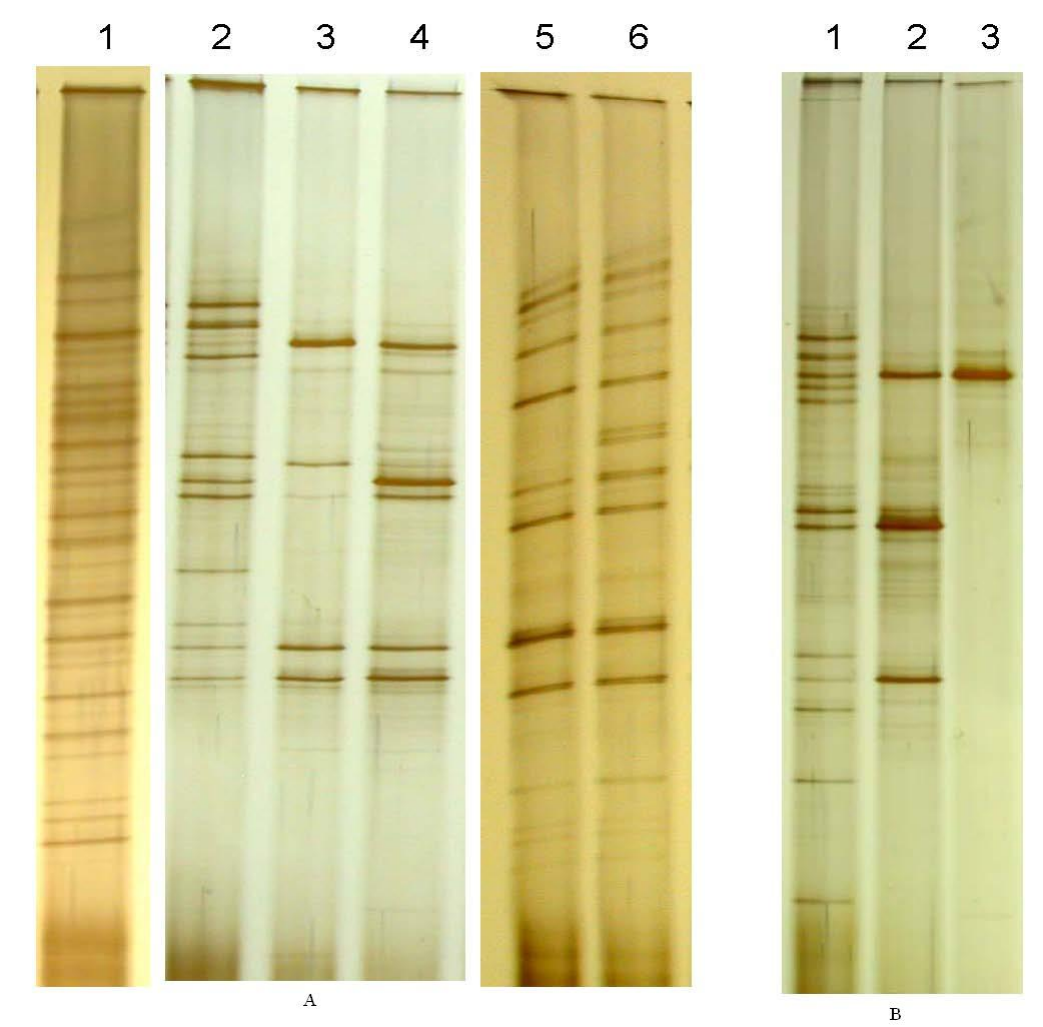
**PCR-DGGE bacterial profiles showing the richness of bacterial populations **. **A) **Bacterial profiles before and after antibiotic treatments. Lane 1: large intestinal digesta sample (LIC); Lane 2: start culture that was the first subculture from the digesta (LIC) before lincomycin treatment; Lanes 3 and 4: same start culture after the treatment with lincomycin at 60 and 30 μg ml^-1^, respectively; Lanes 5 and 6: same start culture after the treatment with tylosin at 80 and 40 μg ml^-1^, respectively. **B) **Changes of PCR-DGGE bacterial profiles through the selection by antibiotics and AIM+CecExt medium. Lane 1: start culture (1^st ^subculture from the digesta) before antibiotic and AIM+CecExt treatments; Lane 2: the same culture (in Lane 1) after antibiotic and AIM+CecExt treatments; Lane 3: a pure culture of a single colony isolate with DON-transforming activity (Isolate LS-61). Note: Lane 1, lanes 2 - 4, and lanes 5 - 6 of Panel A were from three separate DGGE gels. The migration of their DNA bands was not identical among the different gels.

PCR-DGGE bacterial profiles were used to guide the selection for DON-transforming bacteria in this study. Fig. 4 displays examples to show the effectiveness of PCR-DGGE bacterial profiles in guiding the bacterial selection. The large intestinal digesta sample (Panel A - Lane 1) had many more DNA bands than the start culture (Lane 2) that was a subculture from the digesta, indicating the selective effect of subculturing. It was described above that tylosin had no detrimental effect on either DON transformation or bacterial growth of the start cultures at all tested concentrations. However, the treatment showed little influence over the richness of bacterial populations, as indicated by the similarity of PCR-DGGE bacterial profiles before and after tylosin treatment (Panel A - Lanes 2, 5, and 6). Thus no further experiments were pursued with the resulting cultures. In contrast to tylosin, treatment with lincomycin significantly reduced the richness of bacterial populations indicated by the reduction of DGGE DNA bands (Panel A - Lanes 2, 3, and 4), but did not affect DON transformation. The resulting cultures were subsequently used for further bacterial selection. Panel B shows the changes in the richness of bacterial populations during the selection process for DON-transforming bacteria. The number of DGGE DNA bands decreased during the process of selection until a single colony isolate was obtained, which demonstrated a single major DNA band in the DGGE gel (Lane 3).

### Identification of DON-transforming bacterial isolates

The sequence similarity analysis of partial 16S rRNA genes (~700 bp) of the 10 isolates with DON-transforming activity indicated that they belonged to four different bacterial groups, Clostridiales, *Anaerofilum*, *Collinsella*, and *Bacillus *(Table 2). Isolates within the same group had sequence similarities greater than 99%. However, isolates located in different groups showed sequence similarities less than 85%. One isolate, named LS-100, had 99% similarity in the partial sequence of 16S rRNA gene compared with that of *Bacillus arbutinivorans*.

**Table 2:** Putative identity of the selected DON-transforming bacterial isolates

		Blast search			RDP Classifier
			
Groups	Isolates	Closest relatives	Accession #	Homology (%)	Closest identification
1	SS-3	Uncultured bacterium clone p-662	AF371567.1	98	Clostidiales order
	LS-61	Uncultured bacterium clone B778	AY984815.1	96	Clostidiales order
	LS-107	Uncultured bacterium clone B778	AY984815.1	96	Clostidiales order

2	LS-72	Unidentified bacterium clone CCCM8	AY654968.1	99	*Anaerofilum *genus
	LS-83	Unidentified bacterium clone CCCM8	AY654968.1	99	*Anaerofilum *genus

3	LS-94	*Coriobacterium *sp. EKSO3	AJ245921.1	97	*Collinsella *genus
	LS-117	*Coriobacterium *sp. EKSO3	AJ245921.1	97	*Collinsella *genus
	LS-121	*Coriobacterium *sp. EKSO3	AJ245921.1	96	*Collinsella *genus
	LS-129	*Coriobacterium *sp. EKSO3	AJ245921.1	96	*Collinsella *genus

4	LS-100	*Bacillus arbutinivorans*	AF519469.1	99	*Bacillus *genus

### Stability of DON-transforming activity of the isolates during subculturing

The stability of the 10 bacterial isolates in DON transformation during subculturing in L10 broth was examined. Six out of the 10 isolates retained 100% of the activity over the six passages of subculturing (Table 3). However, the activity of isolates LS-117 and SS-3 disappeared after 3 to 4 passages of the subculturing. In contrast, isolates LS-129 and LS-121 initially demonstrated partial activity of DON transformation, but their activity was fully developed (100% transformation of DON to DOM-1) through 2 to 3 passages of subculturing. Isolate LS-100 was transferred for four additional passages. It retained full activity during the additional passages regardless of the presence or absence of DON in the medium.

**Table 3:** Activity in transforming (%) DON to DOM-1 of subcultures of DON-transforming bacterial isolates

Isolates	Sub-1	Sub-2	Sub-3	Sub-4	Sub-5	Sub-6
SS-3	100	77.9	14.3	2.1	0	0
LS-61	100	100	100	100	100	100
LS-72	100	100	100	100	100	100
LS-83	100	100	100	100	100	100
LS-94	100	100	100	100	100	100
LS-100	100	100	100	100	100	100
LS-107	100	100	100	100	100	100
LS-117	47.5	9.2	1.5	0	0	0
LS-121	56.2	7.8	18.9	100	100	100
LS-129	31.6	43.4	100	100	100	100

## Discussion

The application of microbial transformation of mycotoxins has been largely limited in the past by the unavailability of microbial agents. Although the animal intestine has been frequently shown to be a habitat for bacteria, isolation of pure bacterium with transformation capability has remained a great challenge due to the large number of microorganisms (10^11-12 ^cells ml^-1 ^in the large intestine) in the animal intestine and the complexity of intestinal microbiota. He *et al. *[12] described a high activity of mixed microorganisms from the chicken large intestine in transforming DON. However, they were unable to purify the microorganisms. The present study describes an approach using PCR-DGGE bacterial profiles to guide the selection of DON-transforming bacteria through the use of conventional microbiology techniques. The integration of PCR-DGGE bacterial profiling into the selection has significantly improved our efficiency in selecting desired bacteria. With this integrated approach, a microbial community with DON-transforming activity was effectively reduced to only 10^3 ^CFU ml^-1 ^from the level of 10^11-12 ^CFU ml^-1^. The approach has provided a success rate of approximately 5% (10 positives out of 196 examined). This is much more efficient than traditional blind screenings. For example, only one active colony was obtained after screening thousands of colonies using a traditional approach alone in a previous study [13]. Thus, the approach developed in the present study can be used as a common strategy for bacterial selection. This is the first report of a successful isolation of pure cultures of DON-transforming bacteria from the chicken intestine using a DNA bacterial profiling-guided selection. Also, the research has clearly demonstrated that one of the selected isolates (LS-100) is highly consistent and potent in the transformation of DON and transformation of other trichothecene mycotoxins [20]. It is worth pointing out that isolate SS-3 was selected from the small intestine. Considering that this isolate may offer an advantage in colonizing the small intestine, a region with high physiological significance for animal nutrition, more studies are warranted. In summary, the isolation of pure cultures of DON-transforming bacteria has provided a good opportunity for biotransformation research and applications including physiology underlying the transformation and development of microbial or enzyme products for field application.

The sequence data of partial 16S rRNA genes indicate that the 10 selected isolates with DON-transforming activity belong to four bacterial groups. This diversity may give the host an advantage to ensure the consistency of DON-transformation in the chicken intestine [5,12,14]. Despite taxonomic distance between the isolates, they share similar DON transformation function. During the *in vitro *selection with DON as the sole carbon source in the mineral medium (AIM), DON-transforming bacteria were unable to utilize DON as a source of carbon and energy, and therefore there was no effect of enrichment. However, the desired bacteria were enriched when the nutritional requirement was met, evidenced by both *in vivo *and *in vitro *enrichment. This suggests that DON-transforming bacteria may have an advantage in competition in the intestinal environment when DON is present. Furthermore, all the isolates demonstrated the same function of transforming DON to DOM-1 by deepoxidation. Isolates SS-3 and LS-100 have been further studied and shown to degrade other trichothecene mycotoxins by deepoxidation and/or deacetylation [20]. The results are in agreement with the report by Fuchs *et al. *[19], in which pure cultures of *Eutacterium *sp. isolated from the rumen have been studied. It is unclear at present if all the isolates have an identical enzyme or isoenzymes for their DON-transforming activity. Purification and characterization of the enzyme(s) and cloning of the genes encoding the enzymes will lead to a clarification.

## Conclusions

The use of PCR-DGGE guided microbial selection in this study has significantly increased the efficiency for isolating DON-transforming bacteria. The obtained bacterial isolates were able to detoxify DON, which allows further studies for both basic research and application in biotransformation of this mycotoxin.

## Methods

### Culture media

L10 broth [21] amended with 10% rumen fluid was used for culturing chicken intestinal microbiota and L10 agar was used for plating and colony screening. The anaerobic incubation medium (AIM) [12] amended with 10% chicken cecal digesta extract (CecExt) was used for selection of DON-transforming microorganisms. The CecExt was prepared by adding 10 g cecal digesta into 90 ml distill water. The resulting mixture was shaken at 110 rpm at 22°C for 30 minutes and then the supernatant recovered from the mixture was filtrated through a filter (Corning Inc., Corning, New York, USA) with the pore size of 0.22 μm. The media of MRS [22], RB [23], VL [24], and DAM [25] were tested for the selection of DON-transforming bacteria.

### Sample collection and microbial cultures

Intestinal digesta was obtained from Leghorn hens. The chickens were housed on floor with free access to water and a layer diet. All research procedures for using chickens complied with the University of Guelph Animal Care Committee Guidelines. To collect digesta samples, the chickens were euthanized by cervical dislocation and their intestines were removed, placed in plastic bags, and immediately brought into an anaerobic chamber (Coy Laboratory Products Inc., Grass Lake, Michigan, USA) with atmosphere of 95% CO_2 _and 5% H_2_. Digesta was removed from the small and large intestine of individual birds and kept separately for selecting bacteria. The crop content was also collected and each sample was generated by combining the crop content from three chickens in the same treatment group. Microbial cultures were established by adding 0.2 g digesta into 1 ml L10 broth and incubated at 37°C for 72 hrs in the anaerobic chamber. This incubation condition was used throughout all experiments unless described otherwise. Microbial subcultures were obtained from inoculation of a fresh medium with 10% initial culture followed by incubation. DON (100 μg ml^-1^) was included in the media (broth) for all experiments unless otherwise indicated.

### DNA extraction, PCR amplification, and DNA sequence analysis

QIAamp^® ^DNA Stool Mini Kit (QIAGEN Canada, Mississauga, Ontario, Canada) was used to extract genomic DNA from digesta or mixed microbial cultures following the manufacturer's instructions. Qiagen DNeasy Tissue Kit was used to extract genomic DNA from pure cultures of bacterial isolates.

The 16S rRNA genes were amplified from genomic DNA of the isolates by PCR using eubacterial primers F8 (5'-AGAGTTTGATCCTGGCTCAG-3') and R1541 (5'-AAGGAGGTGATCCAAGCC-3') as described previously [26]. PCR amplicons were sequenced using primer 16S1100r (5'-AGGGTTGCGCTCGTTG-3'). Partial 16S rDNA sequences corresponding to *Escherichia coli *16S rRNA bases 300 to 1050 were compared with the GenBank, EMBI, and DBJI nonredundant nucleotide databases using BLAST analysis. The sequences were also submitted to Ribosomal Database Project (RDP) Classifier for identification of the isolates.

### PCR-DGGE bacterial profile analysis

The V3 region of the 16S rRNA genes (position 339 to 539 in the *E. coli *gene) of bacteria was amplified using primers HDA1-GC (5'-**CGC CCG GGG CGC GCC CCG GGC GGG GCG GGG GCA CGG GGG G **AC TCC TAC GGG AGG CAG CAG T-3'; the GC clamp is in boldface) and HDA2 (5'-GTA TTA CCG CGG CTG CTG GCA C-3') as described by Walter *et al*. [27]. PCR reaction mixtures (50 μl) contained 1× PCR buffer (ThermoPol reaction buffer, New England Biolabs, Inc., Pickering, Ontario, Canada), 200 μM of each dNTPs, 0.5 μM of each forward and reverse primers, 4% (v v^-1^) dimethylsulfoxide (DMSO), 2.5 units of Taq polymerase (New England Biolabs, Inc.), and an appropriate amount of template DNA. The 1× PCR buffer (pH 8.8) is composed of 10 mM KCl, 10 mM (NH_4_)_2_SO_4_, 20 mM Tris-HCl, 2 mM MgSO_4_, and 0.1% (v v^-1^) Triton X-100. PCR amplification program consisted of preheating at 94°C for 4 min and 30 cycles of denaturing (94°C, 30 sec), annealing (56°C, 30 sec), and extension (72°C, 2 min) followed by final extension at 72°C for 10 min. The DGGE analysis of PCR amplicons was performed using the Bio-Rad DCode Universal Mutation Detection System (Bio-Rad Canada, Mississauga, ON, Canada). The amplicons were separated in 10% polyacrylamide (acrylamide/bisacrylamide 35.7:0.8) gels containing a 35 to 65% gradient of urea and formamide increasing in the direction of electrophoresis. A 100% denaturing solution consisted of 7 M urea and 40% (v v^-1^) deionized formamide. The electrophoresis was conducted in 1× TAE buffer with 100 V at 60°C for 16 hr. DNA bands in gels were visualized by silver staining [28]. The number of DNA bands, including the presence and density, were used to determine the richness of bacterial populations. The BioNumerics software (version 3.0, Applied Maths, Sint-Martens-Latem, Belgium) was used for similarity analyses of the profiles as described previously [29].

### Extraction and quantification of DON and DOM-1

The detailed procedures of DON extraction and quantification were described previously [20]. Briefly, DON was extracted from a bacterial culture using acetonitrile. The extracts were dissolved in methanol/water (1:1 in volume) and filtered through a C18 SPE cartridge (Phenomenex, Torrance, CA, USA). The extracts were analyzed for DON and DOM-1 by injecting 20 μl aliquot into an Agilent Zorbax Eclipse XDB-C18 column (4.6 × 150 mm, 3.5 μm) followed by detection with a ThermoFinnigan SpectraSystem UV6000LP detector and a ThermoFinnigan LCQ Deca MS spectrometer. The MS was operated in the positive APCI mode. DON or DOM-1 were quantified on the basis of integrated peak areas using absorbance units (UV) at 218 nm or multiple ion counts (MS) at m/z 231, 249, 267, 279, and 297 for DON and m/z 215, 233, 245, 251, 263, and 281 for DOM-1. These values were compared against UV and MS values taken from calibration curves of authentic DON and DOM-1. The ratio of DON to DOM-1 transformation was calculated as:

Transformation ratio = (DOM-1)/(DON + DOM-1) × 100.

### Selection of DON-transforming bacterial isolates

An integrated approach was designed to select DON-transforming bacterial isolates from intestinal digesta samples (Fig. 2). The approach consisted of *in vivo *enrichment and *in vitro *selection with combined use of conventional microbiological selection strategies and PCR-DGGE bacterial profiling techniques. The activity of DON transformation and the richness of bacterial populations were the two criteria used for the selection. During the entire process for selecting DON-transforming bacteria, PCR-DGGE bacterial profiles were analyzed after each treatment and used to guide the selection of the bacteria. When a sample exhibited a high activity of DON transformation and a significant reduction in the richness of bacterial populations (species) after a particular treatment, the sample was then used for further bacterial selection.

The first subcultures from LIC and SIC digesta samples of the chickens fed DON-contaminated wheat in the *in vivo *experiment (Step 1 in Fig. 2) were used as the start cultures (Step 2 in Fig. 2) for the bacterial selection. The LIC start cultures were initially subjected to the antibiotic treatment (Step 3 in Fig. 2). The resulting cultures through the antibiotic selection were then grown in the AIM+CecExt medium to further eliminate unwanted bacteria (Step 4 in Fig. 2). The SIC start cultures were, however, treated only with AIM+CecExt before single colony isolation (Step 5 in Fig. 2).

#### *In vivo *enrichment

Twelve 69-week-old Leghorn hens were divided into 2 groups. One group (6 chickens) was fed a layer diet supplemented with clean wheat, the other group with contaminated wheat containing 10 ppm (μg g^-1^) DON. The trial lasted for two weeks with digesta samples collected on day 7 and 14, respectively.

#### Antibiotic-based selection

Bacitracin, carbadox, gentamicin, lincomycin, penicillin G, salinomycin, streptomycin, tylosin, vancomycin, and virginiamycin at different concentrations (Table 1) were used to suppress unwanted bacterial populations during the *in vitro *selection for DON-transforming bacterial isolates. The antibiotics were initially tested individually, and then in different combinations for their effect on the activity of DON transformation and on the richness of bacterial populations determined by the PCR-DGGE analysis. The concentrations of each antibiotic were selected based on their level in feeding practice for prophylactic use in food animal production. The tested antibiotics were included in L10 broth during the incubation of microbial cultures for the selection.

#### AIM + CecExt medium-based selection

The AIM+CecExt medium offered an advantage in retaining the activity of DON transformation with a minimum support for the growth of bacterial populations. The medium was therefore used after the antibiotic selection to further reduce unwanted bacterial populations. Briefly, the cultures completely transformed DON to DOM-1 through the antibiotic selection were diluted 10-fold in series in AIM + CecExt followed by incubation for 72 hrs and examined for the activity of DON transformation. The cultures with a highest level of dilution and full activity of DON transformation were further diluted in AIM+CecExt. An aliquot of 0.1 ml of each dilute was plated onto L10 agar for screening colonies and mixed with 0.9 ml of L10 broth to grow a culture for PCR-DGGE bacterial profiles, respectively. Sixty eight single colonies from SIC and 128 colonies from LIC were screened for their ability to transform DON to DOM-1.

## Authors' contributions

HY designed and carried out experiments for bacterial selection, performed data analysis and interpretation, and coordinated routine research activities. JG and TZ conceived the research and contributed to experimental design and interpretation of results. CY and HZ performed quantitative analysis of DON transformation. XS performed PCR-DGGE bacterial profile analysis. XZL performed the subculturing experiment of single colony isolates. RT and RY developed a protocol for effective extraction of DON for chemical analysis. HY and JG prepared the manuscript. All authors read and approved the final manuscript.
